# Surgery on the battlefield: Mobile surgical units in the Second World War and the memoirs they produced

**DOI:** 10.1177/09677720211012190

**Published:** 2021-06-03

**Authors:** Katherine M Venables

**Affiliations:** 1 St Cross College, 6396 University of Oxford, Oxford, UK

**Keywords:** Second World War, mobile surgical units, medical memoir, literature

## Abstract

In the Second World War, there was a flowering of the battlefield surgery pioneered in the Spanish Civil War. There were small, mobile surgical units in all the theatres of the War, working close behind the fighting and deployed flexibly according to the nature of the conflict. With equipment transported by truck, jeep or mule, they operated in tents, bunkers and requisitioned buildings and carried out abdominal, thoracic, head and neck, and limb surgery. Their role was to save life and to ensure that wounded soldiers were stable for casualty evacuation back down the line to a base hospital. There is a handful of memoirs by British doctors who worked in these units and they make enthralling reading. Casualty evacuation by air replaced the use of mobile surgical units in later wars, throwing into doubt their future relevance in the management of battle wounds. But recent re-evaluations by military planners suggest that their mobility still gives them a place, so the wartime memoirs may have more value than simply as war stories.

## Introduction

‘In the various military histories of the war in South-East Asia, the intimate details of the fighting are adequately recorded but no more than passing mention is made of the part played by the Medical Services, in particular, the Mobile Surgical Units.’ The quotation is from John Baty's^1^ *Surgeon in the Jungle War*, published in 1979, decades after the Second World War ended in 1945 and when there had been plenty of opportunity for ‘adequate records’ to be written. In the Royal Army Medical Corps (RAMC) Baty commanded the Seventh Indian Mobile Surgical Unit (7 IMSU) from May 1943 during the campaign against the Japanese in the Arakan region on the coast of Burma. My father, Harry Walker, was an anaesthetist who joined a different unit, 5 IMSU, in May 1944 and supported the troops at Kohima and Imphal in Assam, through central Burma to Rangoon, and on to Singapore at the Japanese surrender in 1945. His unit then treated Javanese civilians injured in the Indonesian revolution and he returned to England in 1946. I am currently working on a reconstruction of his wartime experience.

It is perhaps surprising that there was no post-war evaluation of mobile surgical units (MSUs), given that they were one of several important changes to the organisation of medical services for casualty evacuation that were introduced on the recommendation of the wartime Hartgill committee in 1941.^2^ The reason may have been an assumption that MSUs would be superfluous in future conflicts when improved air evacuation would move wounded men rapidly to well-equipped surgical hospitals away from the front. Indeed, rapid casualty evacuation by helicopter was a feature of the Korean War (1950–1953) and Vietnam War (1964–1975) as portrayed in Richard Hooker's 1968 novel, Robert Altman's film M*A*S*H, and the spin-off television series.^3–5^ ‘Richard Hooker’ is the pen-name for the collaborating authors Richard Hornberger, a former US Army surgeon, and W C Heinz.

But, in his 1993 thesis, Patillo studied the 1940s Burma campaign experience in order to explore ‘problems with rapid deployment and ground mobility’ of American mobile hospitals in the 1980s and 1990s. During the American invasion of Grenada in 1983 the field hospital that was deployed proved to be less mobile than was required and, in the first Gulf War of 1990–1991, the large numbers of nursing staff and beds of American field hospitals ‘adversely affected mobility.’ Many medical units created *ad hoc* forward surgical teams and Patillo concluded that the MSU had ‘re-emerged on the modern battlefield.’^6^

The issue of how modern armies should provide forward surgical capability remains moot. A 2015 article from the Canadian Armed Forces emphasises the role of ‘damage-control surgery’ in special operations in ‘austere environments with fewer personnel and a smaller logistical footprint than conventional operations.’^7^ The authors of a 2020 paper comment that ‘truly austere circumstances’, with little opportunity for air evacuation, prevail in many conflicts today, as in the Second World War. These conditions are also replicated in natural disasters and well-prepared forward military surgical teams, with standardised equipment and procedures, are vital.^8^ There is current interest in how these teams should be organised and, for example, Chinese military doctors in 2018 reviewed the literature on their composition and work.^9^ Memoirs by doctors from a war many decades ago may therefore be relevant for today’s military medical planners.

## Origins of the mobile surgical unit

Along with the MSU, this article mentions two other kinds of mobile unit which carried out surgery in the Second World War: the field ambulance and the mobile hospital. A field ambulance, as Donaldson^10^ writes succinctly in his memoir, is not a vehicle. ‘Rather, it’s a small mobile field hospital with a staff of about 50 operating just behind the front line […] Each of the three Brigades in a Division has its field ambulance.’ Base hospitals were large general hospitals with up to 1000 or more beds and a full complement of support services, housed in tents, huts and available buildings. For example, 21 British General Hospital, where my father served when he was first in India, started life in France for the first few months of 1940, was evacuated from Dunkirk when the Germans invaded, then spent the next two years in Leeds, Sussex and Perthshire before moving to India in June 1942. In India, the hospital moved from Secunderabad to Trimulgherry, Jhansi, Dinapore and Calcutta before disbanding in Madras in 1946.^11^

The MSU is generally accepted to have had its definitive origins in the Spanish Civil War of 1936–1939. In the previous major European conflict, the First World War, the aim of casualty evacuation was not to take the surgeon to the casualty but to remove the injured from the battlefield to a hospital out of range of artillery fire. Because most of the fighting fronts were essentially static and air warfare was in its infancy, large hospitals could be set up in tents or buildings in a safe environment several miles from the fighting, to which casualties could be evacuated by ambulance train or horse- or motor-ambulance. Although several MSUs were equipped they were set up too late in the war to be deployed to any effect.^6^

However, the potential of MSUs was clear even in the First World War. For example, the work of an Australian ‘advanced operating unit’ in Palestine was described in a 1940 essay by Wade.^12^ This unit was deployed in 1917 during cavalry operations in Beersheba and elsewhere. It used a ‘surgical operating car’ to carry personnel and equipment and to provide electrical power. Quoting the *Official History* of the First World War, Wade observed that, ‘the equipment required […] was not necessarily either bulky or elaborate, and […] surgeons could co-operate advantageously with the various field ambulances [which] welcomed the arrangement by which they were relieved of the onerous responsibilities of carrying out the surgical treatment of those patients who required serious and prolonged operative treatment.’

The Spanish Civil War was more mobile than the First World War and the extensive use of aerial bombing by the Nationalists and their allies, the Nazi regime in Germany, meant that all medical units were now vulnerable and aggregations of transport or casualties could become targets. Traditional military hospitals, with their large amounts of equipment and personnel, were cumbersome and slow to move. The familiar saying that war forces innovation proved correct in this setting. One paper has pointed out that at the onset of the Civil War most Spanish military doctors defected to the Nationalists, leaving Spanish civilians and young foreigners as the doctors working with the Republicans. They had a pressing need to improvise and no commitment to the *status quo*.^13^ Two elements became important: the mobile field hospital (or ‘field ambulance’) and the MSU (sometimes called a field surgical unit or forward surgical unit). The MSU consisted of two or three doctors and around 10 or 12 support staff, including nursing orderlies, in self-sufficient units which could run two operating tables. This surgical capability could be deployed flexibly to field hospitals as the military situation dictated.

Two surgeons who worked in the Spanish Civil War published influential books: Trueta's *Principles and Practice of War Surgery*^14^ and Jolly’s *Field Surgery in Total War*.^15^ Both stressed that the patient's outcome was greatly improved by minimising the elapsed time between wounding and surgery. This allowed prompt resuscitation including blood transfusion, wound debridement which reduced the risk of infection, immobilisation of fractures, and life-saving procedures such as tracheotomy or the ligation of blood vessels. The aim was to save life and improve the casualty's chances of surviving evacuation to a well-equipped base hospital where definitive surgery could be carried out at leisure.

Josep Trueta (1897–1977, his forename often anglicised to Joseph) was a Catalan orthopaedic surgeon who was Professor of Surgery in Barcelona during the Spanish Civil War and dealt with thousands of combatant and civilian casualties. He left Spain at the Nationalist victory in 1939 for Oxford, moving back to Catalonia on his retirement. He was a pivotal figure in developments in war medicine, participating in the early penicillin trials, for example. But his major wartime contributions were his studies on war surgery, published as a pamphlet in Catalan in 1939 and later developed into *Principles and Practice of War Surgery* in 1943. In it, he emphasised five points for the treatment of limb wounds: prompt surgical treatment, cleaning the wound, excision of devitalised tissue, drainage, and plaster immobilisation.^14,16^

Douglas Jolly (1904–1983) was a surgeon from New Zealand who, while studying in London, joined a volunteer unit in 1936 and travelled to Spain where he formed a MSU on the Republican side. On the outbreak of the Second World War, he wrote *Field Surgery in Total War*, published in 1940. A recent biographical study published in this journal emphasises Jolly's contributions in the areas of triage, the treatment of abdominal wounds, and wound closure techniques, as well as his advocacy of Trueta's plaster technique for limb wounds.^13^ He proposed an organisational concept called the ‘Three Point Forward System’ with triage as far forward as practicable, which shunted the injured either to No. 1 hospitals close to the front or to No. 2 hospitals further back, prior to onward movement to evacuation hospitals. He concluded that ‘the reduction of the time-lag for the gravely wounded necessitates a reorganisation throughout the system.’^15^

## The Second World War

When the Second World War began, the British Army's casualty evacuation system was still based on the largely static warfare of the First World War.^17^ But the retreat to Dunkirk during the German Blitzkrieg in 1940 demonstrated that medical units were insufficiently mobile; some casualty clearing stations were over-run by the Germans and most lost much of their heavy and bulky equipment. Richard Doll's^18^ articles in the *BMJ* give a young doctor's eye view of the chaos of the retreat. And, in his 1985 *Doctor at Dunkirk*, Ian Samuel, a general practitioner with surgical training, describes the pressures of being out of contact with specialist surgical expertise and adapting a farm kitchen as an operating theatre. ‘I managed to do surgery with quite inadequate surgical instruments,’ he wrote, but he had to abandon many casualties during the retreat and describes giving an overdose of intravenous morphine to ‘impossible’ cases before leaving them behind.^19^

British doctors learned quickly from the Spanish Civil War surgeons. Lectures were delivered.^20^ Hamilton Bailey published the first volume of his *Surgery of Modern Warfare* in July 1941 and the War Office's Hartgill Committee codified the lessons in December 1941.^2,21^ The mobile tank warfare in the Western Desert in North Africa in 1940–1943 showed the need for surgical teams with light logistical demands which could function close to the fighting and move between larger medical units ‘in an ever-changing pattern depending on the tactical situation.’^17^ Furthermore, few British casualties could be evacuated by air until the end of 1942, necessitating treating the wounded close to the front.^22^ By early 1943, the principles of battlefield surgery were widely accepted and in January 1944 the War Office published a short manual for doctors, *A Field Surgery Pocket Book*, and distributed it throughout the year, including to doctors in prisoner-of-war camps. A revised edition appeared in 1950 in response to the Cold War, including the Korean War, and to the Malayan Emergency and other violent independence struggles in the former British Empire.^23,24^

MSUs were not the only type of mobile unit in the Second World War. Field transfusion units, for example, were often co-located with MSUs, and malaria forward treatment units allowed men to recover and return to their units without the delay that was inevitable with evacuation to a base hospital.

## War literature

As John Baty pointed out, the Second World War generated much literature, mainly focussed on strategy and combat. General William ‘Uncle Bill’ Slim published a classic general's memoir from the Burma campaign in 1956 in *Defeat into Victory*.^25^ Other senior officers wrote accounts from the perspective of lower levels of command, notably the leaders of the ‘Chindit’ columns, which penetrated behind Japanese lines: John Masters,^26^ Bernard Fergusson,^27,28^ and Michael Calvert.^29^ The novelist George Macdonald Fraser wrote an important memoir from the viewpoint of an ordinary soldier, *Quartered Safe Out Here*.^30^

These memoirs rarely refer in any detail to medical units, which tend to be taken for granted by their soldier authors. Harrison, in his history of British military medicine in the Second World War, comments that good commanders like Slim recognised the importance of the medical services in maintaining fighting strength and the men’s morale but, for some, they were a disagreeable burden. General Orde Wingate, the instigator of the Chindit forces, for example, insisted that ‘Every man with my force must be a fighting man.’ He wanted ‘no passengers nor Geneva Convention people.’^22^ In a post-war evaluation for the Office of Medical History of the US Army Medical Department, the senior medical officer to the Chindits, William Officer, wrote that communication between fighting officers and medical officers was poor. ‘The habit of looking upon the doctor as a Fifth Columnist likely to blab the merest piece of confidential information which is vouchsafed him, is still all too prevalent.’^31^ The memoirs from MSUs confirm this impression and present the units as reacting to events, rather than having a role in planning. But this distance from strategic responsibility, coupled with their clinical autonomy, appeared to energise some doctors and at times the memoirs have a conspicuously buccaneering tone.

Life in the front line suited the more adventurous doctor. John Vaughan, for example, in his 1988 *All Spirits*, describes an itinerant pre-war life as a ship's surgeon, film unit doctor, and medical officer in colonial outposts, before taking a casualty officer job in the London Blitz. But ‘the pay of even a lieutenant RAMC […] was a princely sum compared with my thirty shillings weekly.’ He joined the 224 Parachute Field Ambulance and volunteered for the glider attack on Pegasus Bridge near Caen as part of the D-Day landings in June 1944. In the last few weeks before the German surrender, he was seconded to the Special Air Service, operating ahead of the main body of troops and seeing Belsen, the link-up with the Russians, and the streams of displaced persons on German roads.^32^

## Battlefield practice

**Figure 1.**
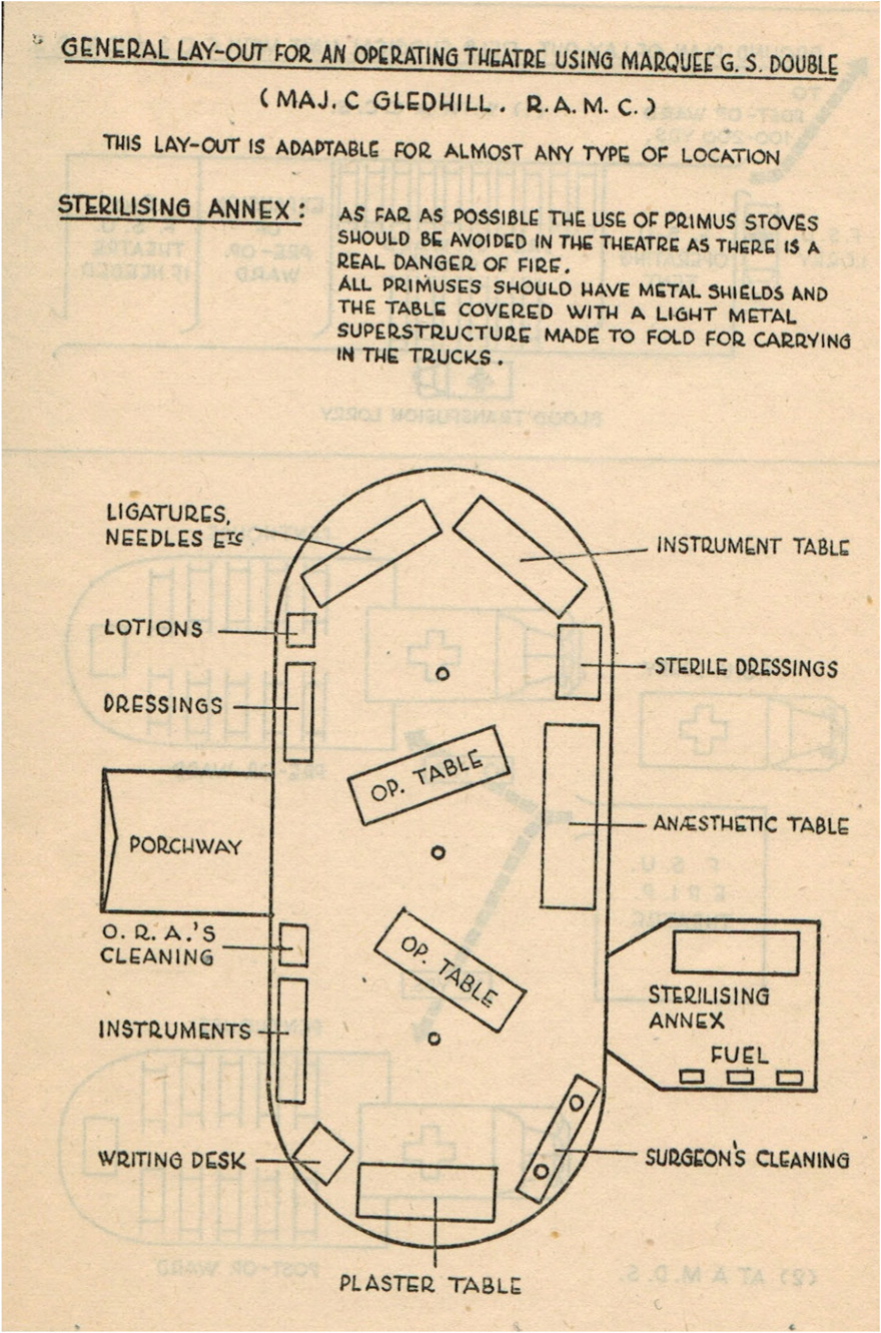
Layout of an operating tent, from the 1944 *Field Surgery Pocket Book*, page 21.^23^

*A Field Surgery Pocket Book* sets out the expectations for MSUs at the time. It starts by reminding the reader that casualties can be grouped as: requiring resuscitation before any surgery or evacuation, requiring immediate operation and holding until fit to move, and fit for evacuation. Units could be deployed ‘as far forward as practicable’ to deal with soldiers needing immediate surgery, or further back in order to operate at greater leisure on evacuated casualties as well as on any local wounded. ‘The wounded may start to arrive late in the evening,’ the manual informs its readers, meaning that attention to the generator and the lighting is vital. It warns that each operation takes, on average, an hour, wounds are frequently multiple, and a surgical team cannot deal effectively with more than 12–16 such cases in 24 hours, even in ideal circumstances. It lays great emphasis on triage and on rapid decision-making, which ‘saves time and more cases will be dealt with and unnecessary or hopeless operations will be avoided. […] Keep fit and get as much rest as possible. Stamina will be severely tested during times of stress. […] Do not waste energy by operating on patients who should be evacuated. Indeed, it may be less hazardous to evacuate a wounded man than to operate.’ This emphasis on standardisation and simplification was quite different from the variations in individual practice that a civilian surgeon expected at the time. Figure 1 shows the *Pocket Book*'s suggested layout for an operating tent.

Most of the injuries the units dealt with were caused by mortar bombs or shell fragments, with machine gun and rifle bullets causing most of the remainder. The most frequent injuries seen were limb wounds, but others, though less common, were often life-threatening and the first priorities for immediate surgery were abdominal wounds, including of the perineum and buttocks, open chest wounds, and severe maxillo-facial and other head wounds, including burns and complicated eye wounds. The units dealt with open limb fractures and complicated soft tissue injuries requiring amputation. The interval after wounding was almost always longer than was usual in emergency surgical practice in civilian life, and sometimes much longer because of enemy action. If evacuation from the fighting had been delayed by days, or even weeks, then patients would arrive with infected wounds, gas gangrene, maggot infestation, or tetanus. In the Burma campaign, the patient's general condition could be compromised further by malaria, vitamin deficiency or dysentery.

Another major difference from civilian practice was the lack of continuity of care. If time and the state of the conflict permitted, the unit might retain a few patients in order to complete their treatment but usually the forward surgeon was only the first in a chain of surgeons back to the base hospital. The primary role of the MSU was to make the patient and his wound safe to travel and as soon as the patient was fit to be moved he was evacuated down the line. The teams were expected to clean wounds of any accessible foreign bodies, debride devitalised tissue, control bleeding by tying off any damaged larger blood vessels, and incise fascial compartments to relieve tension. With rare exceptions, they should not ‘waste time’ searching for foreign bodies, inserting drains, or suturing. Sulphonamide powders and, later, topical penicillin were routinely dusted into the wound. The wound was left open, protected with vaseline gauze, covered with sterile cotton wool and bandaged. Limb wounds were splinted, usually by plastering. Clear and accurate record-keeping was emphasised, so that surgeons in base hospitals understood what the MSU had done.

The manual assumed (though this varied in practice) one surgeon, one anaesthetist, two operating room assistants and seven general duty orderlies who undertook the preparation of patients, plastering, overseeing the generator, ensuring a supply of water, laundry, and equipment sterilising. The units carried portable operating tables and beds and enough equipment, linen and dressings for one hundred cases but ‘the surgeon should be prepared to work […] if necessary without gloves.’ It was soon appreciated that in order to be effective they needed to control their own transport. The units were often paired so that surgical teams could spell each other. The host (usually a casualty clearing station or field ambulance, often also hosting a field transfusion unit and, in Burma, a malaria forward treatment unit) was responsible for additional nursing, feeding patients, and casualty transport. It also undertook the general administration of the MSU, its pay, rations and discipline, and maintained its contact with headquarters. (This feature of the units does not help the researcher; a MSU was not expected to maintain its own unit war diary and so there are only a few of these useful documents in The National Archives.)

## Early writing by doctors

In surveying the Second World War literature by doctors, the first thing to note is that an older generation of doctors wrote many articles and chapters to inform their juniors of their insights they had gained in the First World War and even the Boer wars. The Spanish Civil War experience gave up-to-date advice to the new Army surgeon or anaesthetist. Throughout the conflict, the *BMJ* and *Lancet*, as well as the *Journal of the RAMC*, published articles to inform and educate their readership about technical medical topics relating to the war.

After the War, the medical journals published ‘lessons learned’ articles, such as Wigglesworth's account of casualty evacuation.^33^ The various *Official Histories* were slowly published, including the volumes of the *Medical History of the Second World War*, concluded in 1968 by *The Principal Medical Lessons of the Second World War*.^34^ But, as the military historian John Keegan pointed out in his influential 1976 *The Face of Battle*, historians compiling official histories may achieve ‘the remarkable feat of writing an exhaustive account of one of the world’s greatest tragedies without the display of any emotion at all.’ He contrasted the dryness of some military history with the immediacy of war fiction and advocated a cautious use of memoir, directing historians to investigate contemporaneous letters and journals, leave the archive, visit battlefields, and talk to soldiers. Some exploration of emotions was essential.^35^

A handful of more personal memoirs trickled out during or soon after the war ended. Houghton,^36^ in her analysis of memoirs by Second World War veterans, has pointed out that publication was constrained by paper rationing and, more importantly, by censorship. From 1942 to 1945, the British government banned the publication of service reminiscences. There were a few exceptions but they were heavily censored. Until the mid-1950s serving officers, or veterans who had been involved in wartime intelligence work, were required to submit their manuscripts for a security review.

Perhaps the first memoir of mobile surgery in the Second World War was the 1944 *Burma Surgeon* by the American missionary doctor Gordon Seagrave.^37^ Seagrave created a mission hospital in the China–Burma borderlands in the 1920s. He joined the US Army Medical Corps during the Japanese invasion of Burma in 1942, forming a MSU which accompanied the American General ‘Vinegar Joe’ Stilwell, commanding Chinese forces, on his retreat to India. Seagrave's book fitted well with the contemporaneous American political and military strategy to support China.^38^ He returned to Burma after the War and published several books which raised his hospital's profile and funds for mission activity.

The first British medical memoir including descriptions of mobile battlefield surgery (and one of the best) that my search discovered was published in 1948 anonymously by ‘J A R.’^39^ The author had varied postings after joining the Army, which included surgery in the Western Desert and in a casualty clearing station in Italy. It is structured like a novel, moving from the English Channel before Dunkirk round the Mediterranean and European battlefields to finish with the author in the Channel again, looking at the white cliffs of Dover while talking about his return to civilian life. It incorporates well-written dialogue and descriptions of landscape and operating teams. It is an excellent advertisement for the military surgical life and attracted a Foreword by the Director-General of the Army Medical Services which comments that it ‘is the authentic stuff.’ Although not avoiding difficulties and danger, it is a notably constructive and upbeat account which supports the Foreword's assertions that the medical service in the war was ‘a happy, confident brotherhood’ and that ‘to see an advanced operating centre or casualty clearing station at work during a battle […] gives an unforgettable impression of teamwork at its best.’

John Watts published his memoir in 1955.^40^ Unlike J A R he was a professional soldier, joining up in 1938 and after the War serving in Korea and in a UK military hospital. As a houseman, his chief had told him he would ‘get all the surgery you want’ in the Army because a war was coming, together with a socialist government and ‘you will be better off in a service that you have joined of your own free will.’ Like J A R, Watts worked in mobile units in the Western Desert, Italy and Germany. Like my father, he was part of the British force in Java which attempted to suppress the Indonesian revolution immediately after the Japanese surrender. His account has many practical descriptions and well-argued conclusions about battlefield surgery but written from the perspective of a conservative soldier at the end of Empire. Unlike the books by civilians, his account is peppered with anecdotes from the Mess and whimsical descriptions of the troops and civilians which are of their time but which, to today's ears, sound patronising.

My copy of Thomas Hamilton's 1958 *Soldier Surgeon in Malaya* is a battered paperback, its strident black and yellow cover dominated by the face of a brutal Japanese prison guard. The cover shouts in red capital letters that it is a ‘true and realistic story of a Japanese prisoner of war camp’ but it is not one of the almost voyeuristic accounts of cruelty and deprivation that were published at the time. Instead, it describes mobile surgery during a retreat, as the Japanese made their stunning advances in 1941 and 1942.^41^

## Related life-writing

The accounts of surgery by doctors who were taken prisoner form a special case of wartime medical life-writing which this article does not consider. These books are not so much about the experience of surgery in mobile warfare but of medicine practised under extremely adverse conditions with grossly inadequate resources. They form a sub-section to the large sub-genre of prisoner-of-war memoirs. Some examples are the biography of Bill Frankland by Watkins,^42^ the same authors' article on blood transfusion in this journal,^43^ Aidan MacCarthy's 1979 *A Doctor's War*,^44^ and Glusman's story of four American doctors captured by the Japanese in the Philippines.^45^

This article also excludes memoirs by nurses, such as Angela Bolton,^46^ who did not serve close to the front line in the Second World War, though their accounts provide detail about base hospitals. Some aspects of life in a malaria forward treatment unit in the Burma campaign, as described by Charles Evans,^47^ were similar to life in an MSU.

## Unpublished papers

But much personal experience remained unpublished. Most of the doctors returning from the War were civilians, temporarily recruited to the armed services. For many, life was too busy for writing memoirs. Some returned to pick up roles they had left and there was a government scheme to integrate young doctors back to civilian practice via supernumerary posts. When my father, for example, qualified in Edinburgh in 1942 he had already been a houseman for six months in the wartime accelerated registration scheme. He went into the RAMC on registering and a year later was an anaesthetist in Burma. After returning to the UK in 1946, he was an anaesthetist in a military hospital, worked in general practice to familiarise himself with civilian medicine, and returned to Edinburgh for advanced training before taking up his consultant anaesthetist post in the National Health Service, inaugurated in 1948.^48^ Over the same period, he married and started a family.

Many wartime letters, diaries and contemporaneous case notes were packed away, stored in lofts and cupboards, and surfaced only much later. For example, my father's friend George Blair was a prisoner of the Japanese in Taiwan, an ordeal that affected him profoundly and contributed to his premature retirement. Although he wrote up an account of treating vitamin deficiency from the case notes he had kept, his university felt that the manuscript contained insufficiently rigorous analysis for acceptance as a thesis and the case notes, with his wartime letters, became publicly accessible only when his family deposited his papers in the Wellcome Library after he died.^49^ (One can hope that a university today would respond differently to a former student's extreme and unique experience.)

These archived personal papers throw light in unexpected corners. Women doctors like Molly Newhouse, for example, were recruited into the RAMC but not allowed to work in the front line. There are, therefore, no women surgeons amongst the memoirists considered here. But Newhouse's unusual perspective provides sceptical insights on induction into the Army and on life in a base hospital in India and is also in the Wellcome Library.^50^

## Later writing

More memoirs started to appear as the doctors who had worked in MSUs approached retirement age from the 1970s onwards. Houghton^36^ has observed that the Falklands War of 1982 ‘re-ignited cultural memories’ of the Second World War (and generated its own mobile surgical memoir by Rick Jolly^51^) and that when the various 50th anniversaries came round in the late 1980s and 1990s, publishers were ‘inundated’ with manuscripts by veterans. She has pointed out that memoirs written after the event are composed and published in a social and cultural context that has developed post-war and in which the war has acquired a particular narrative. She also observed that memoirists are self-selected.

While acknowledging Houghton's comments, the medical accounts nevertheless provide a picture of battlefield surgery. The first of these later memoirs that my search discovered was Geoffrey Parker's *The Black Scalpel*, published in 1968.^52^ Already a senior surgeon when he joined the RAMC, Parker became bored with life in a military hospital, took courses in parachuting, small arms and unarmed combat, and applied for a post in a MSU. In the 24th Forward Surgical Unit in North Africa and Italy, he had ‘absolutely magnificent’ equipment and ‘the most brilliant anaesthetist with whom I have ever been privileged to work.’ After a period of illness with jaundice he was approached by a colonel in the Special Operations Executive and parachuted into occupied France, improvising hospitals in a school and a barn with the help of medical students and operating without an anaesthetist. At times he functioned as the leader of a Resistance band, armed with a Colt revolver, combat knife and Tommy gun, and he was later decorated by the French government.

Baty's^1^ 1979 *Surgeon in the Jungle War* was based on operation logbooks and contemporaneous diaries. He describes his medical colleagues: an anaesthetist who acted as second in command (‘we were very much in each other's pockets’) and a Sikh junior doctor who assisted at operations and spoke many dialects, an invaluable skill in the multi-national, multi-lingual 14th Army. His British corporal ran the operating theatre, equipment and supplies. There were Indian and British nursing orderlies and an Indian driver, *dhobi* and sweeper. The mobility and autonomy of his unit give an entrepreneurial flavour to some of Baty's stories. They improvise equipment repairs using bamboo; his corporal ‘liberates’ equipment from the various field ambulances to which they are attached; they prefer to attach themselves to Indian units because their cooks prepare the best food. Baty confronts the commander of a casualty clearing station who wants to over-rule him and writes to New Delhi suggesting improvements in vehicles, equipment and tents. And, overall, the book is full of lively anecdotes about life and surgery. His book also contains sketches of his temporary operating theatres in jungle *bashas* and photographs of his team which, unfortunately, cannot be included in this article because of the *Journal*'s space constraints.

As the doctors who had served in the Second World War aged, biographies, rather than memoirs, appeared, such as Hall’s^53^ biography of the much-decorated doctor Martin Herford in 1995 and Lambourn's^54^ record of his father's life in 2006. After volunteering in 1940 for the Finnish Aid Bureau, whose leaders turned out to be ‘chancers and crooks,’ Martin Herford was briefly in the Royal Air Force before transferring to the RAMC because ‘the conditions under which the MOs and their orderlies worked were as perilous as those endured by the troops.’ His biography illustrates the importance of the competent organisation of surgical services, as much as surgery itself. In Greece and Yugoslavia, he organised casualty evacuation by train, travelling to Crete and Egypt before joining the campaigns in the Western Desert, and then Sicily and Italy. He commanded a field ambulance in the D-Day landings and was taken prisoner while ferrying supplies to the wounded at Arnhem. He set up a prison hospital for British casualties before escaping and returning to his unit.

Stanley Aylett's *Surgeon at War* was published posthumously as late as 2015, edited by his daughter, but the primary memoir on which it is based dates back to a private publication in 1979. A former surgical registrar and ship's surgeon, Aylett was part of the retreat to Dunkirk and then of the desert war in North Africa before commanding the 14th Field Surgical Unit as part of the D-Day landings, and then in Germany and Denmark. His daughter summarises thus:

In the midst of the worst carnage, surgeons would be operating for seventeen-hour stretches, followed by time in the wards, for days on end, making hundreds of quick decisions without the benefit of diagnostic aids. Then, three hours to pack up, a few hours sleep, a journey through devastation, three hours in which to set up, and it would all start again.

As the children of these battlefield surgeons themselves age, Aylett's first-person account of the work of the MSUs may well be among the last to be published.^55^

While accounts by surgeons who served in MSUs are themselves rare, my search has failed to find any memoirs by anaesthetists. Nor have I identified any published by non-commissioned officers, the medical orderlies who worked in these units.

## Images of mobile surgical units

**Figure 2.**
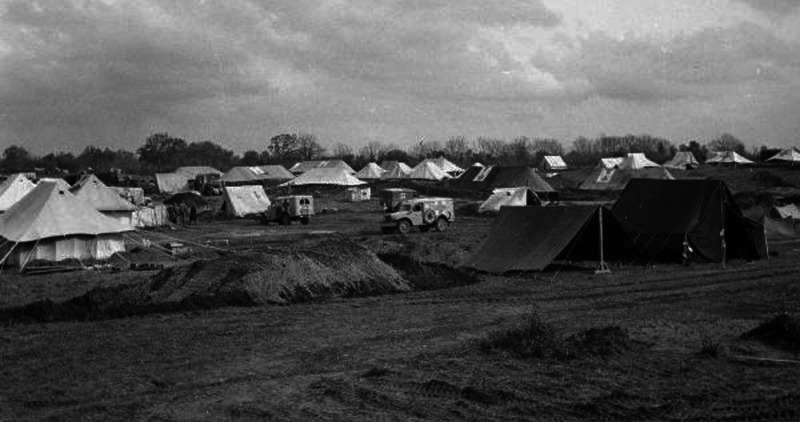
A general view of the tented medical complex, including 36 Field Surgical Unit, at Anzio, Italy, 1944. © Imperial War Museums (NA 12424).

**Figure 3.**
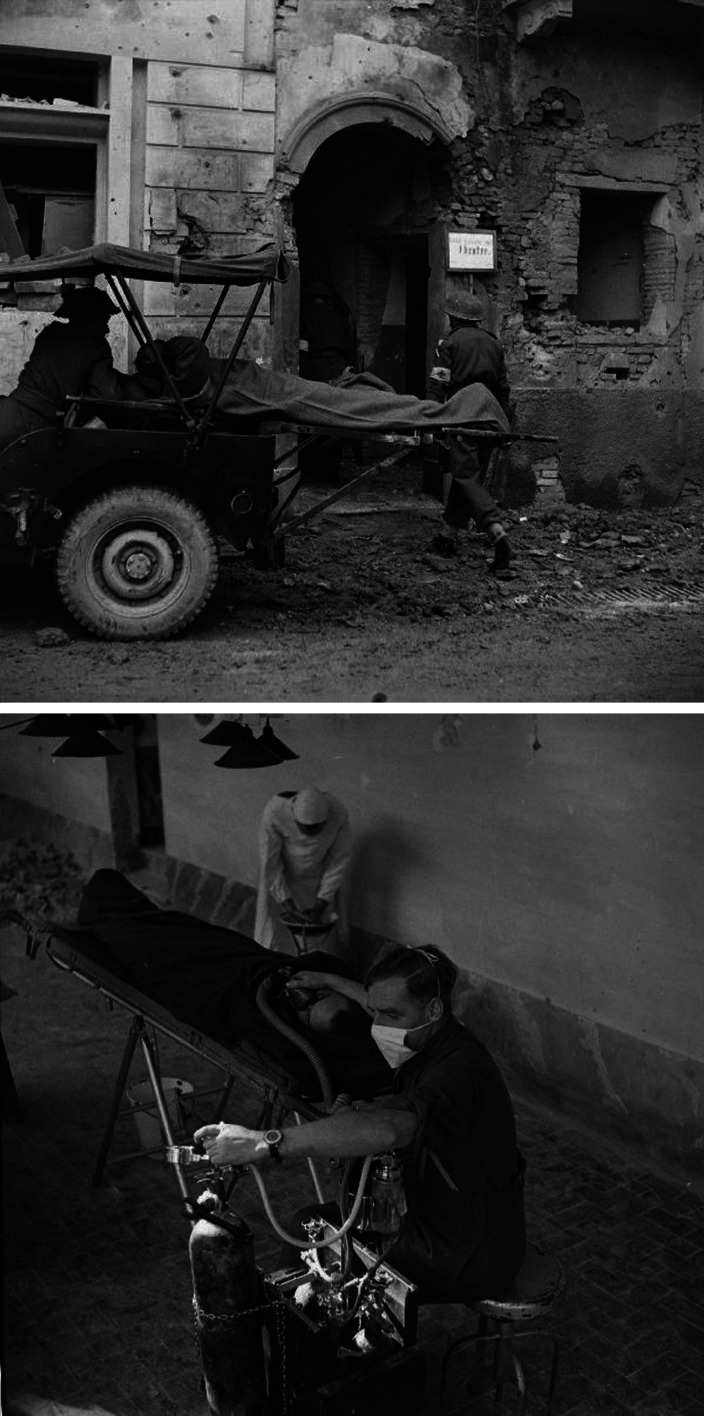
Surgery in an abandoned building under shellfire near Lanciano, Italy, December 1943. Upper panel: Bringing in the casualty © Imperial War Museums (NA 10233). Lower panel: Major Grace gives the anaesthetic © Imperial War Museums (NA 10222).

**Figure 4.**
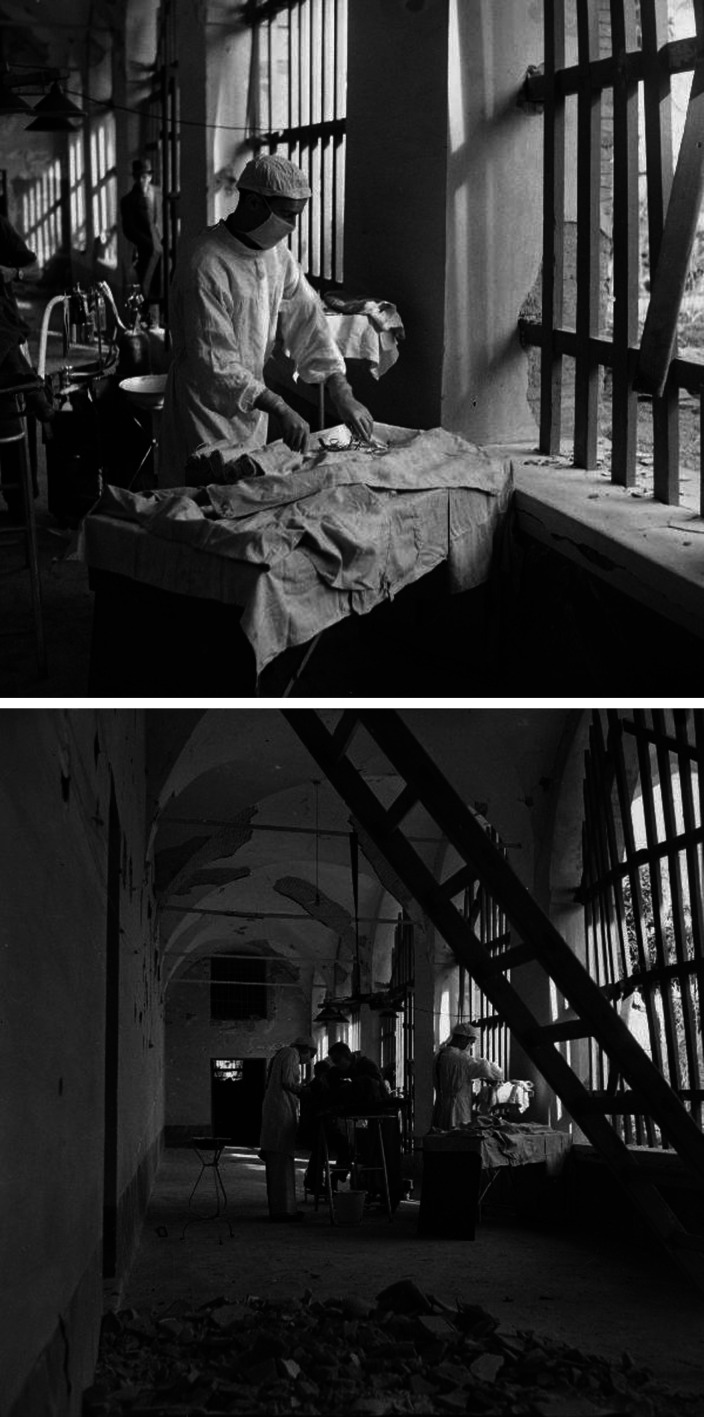
Surgery in an abandoned building under shellfire near Lanciano, Italy, December 1943. Upper panel: Corporal Luxford prepares the instruments © Imperial War Museums (NA 10220). Lower panel: Major Swinney operates © Imperial War Museums (NA 10223).

The War Office and accredited news organisations sent photographers to the Second World War fronts. Among their images of casualties in regimental aid posts or rehabilitation in the base hospitals are a few photographs of MSUs. Figure 2 shows a general view of the tented medical complex, which included 36 Field Surgical Unit, at Anzio, Italy, 1944. The Imperial War Museum archive contains a remarkable sequence of photographs (NA 10218 – NA 10234) of a MSU operating under shellfire in an abandoned building in Italy in 1943. Figures 3 and 4 show selected images from that sequence.

Some wartime photographs have not been digitised, and hence their images do not appear in online searches, so it is possible that there are more photographs of battlefield surgery in the various image collections. In the publicly accessible collections, I have not been able to identify any photographs from MSUs in the Far East, although there are a few casualty evacuation. The only photograph I possess of my father in his operating tent in Burma is from March 1945. He wrote on the back ‘Note operating light made from a Jap “Zero” wing. Cpl. Markwick in picture, also clock from a Burmese house. Were shelled here. Damage – one scraped back from jumping into a trench!’ Camera film and photographic paper were scarce in the War and this is a small print whose content is confusing unless you know what you are looking for. Many other similar photographs of wartime operating theatres must have been discarded by their inheritors in favour of portraits and other more comprehensible images.

## Conclusions

Memoirs by surgeons are popular, as the success of Henry Marsh's^56^ *Do No Harm* exemplifies. The observers of conflict write compelling literature, such as the Vietnam War photographer Michael Herr's^57^ *Dispatches*, or *Contact Wounds* by Jonathan Kaplan,^58^ a surgeon who has worked for disaster relief organisations. Battlefield surgery, likewise, has a heroic, intrepid character that lends itself well to life-writing, both memoir and biography. The best of the Second World War medical memoirs are as readable as good fiction and offer as many empathetic insights into the human condition.

These memoirs also add to our understanding of the evolution of military surgery. After the War, multi-volume official medical histories were published by the governments of the United Kingdom, Canada, Australia, New Zealand, and India. The motto for their concluding volume,^34^ *Respice, Prospice* (look back, look forward), could also be applied to the memoirs I describe in this article. Memoirs by the participants in war are important primary historical documents, along with their journals and letters. They may be subjective and individual, but they also offer rich, granular detail which more conventional histories cannot match. As the military historian John Keegan wrote, allowing combatants ‘to speak for themselves’ is ‘not merely a permissible but, when and where possible, an essential ingredient of battle narrative and battle analysis.’^35^ In the same way, the medical memoirs explored in this article add detail to the official medical histories and are an essential ingredient in the history of the work of these units in the Second World War.
